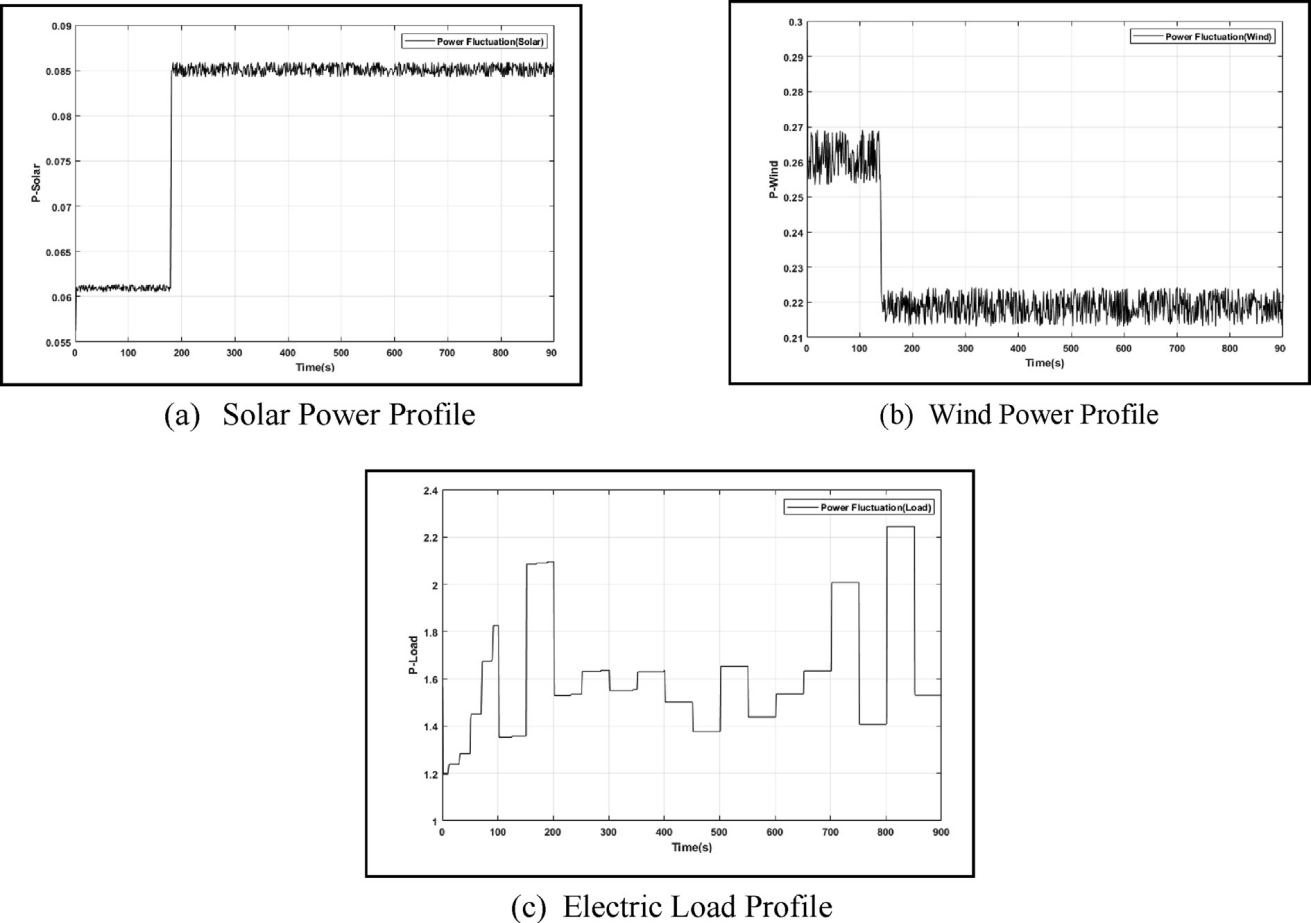# Frequency control of nuclear-renewable hybrid energy systems using optimal PID and FOPID controllers

**DOI:** 10.1016/j.heliyon.2022.e11770

**Published:** 2022-11-21

**Authors:** Riyad Hasan, Md Shafakat Masud, Nawar Haque, Muhammad R. Abdussami

**Affiliations:** a Department of Nuclear Science and Engineering, Military Institute of Science and Technology, Dhaka, Bangladesh; b Department of Electrical and Electronic Engineering, East West University, Dhaka, Bangladesh

**Keywords:** PID controller, FOPID controller, Microgrid, N-R HES, Metaheuristic algorithm, ABC, TLBO

## Abstract

This paper investigates the applications of Proportional-Integrator-Derivative (PID) and Fractional Order PID (FOPID) controllers in Nuclear-Renewable Hybrid Energy Systems (N-R HESs). The N-R HES is a recent technology in the area of decarbonized energy systems. N-R HESs are expected to contribute immensely to providing carbon-free and sustainable energy infrastructure in the upcoming days. It is also anticipated that system resiliency will be the primary concern when nuclear reactors are incorporated with intermittent renewable energy resources. Therefore, in this research, the authors intend to evaluate the compatibility of two classical controllers, PID and FOPID, to ensure the stability of N-R HESs. The N-R HES of this paper consists of different energy sources, such as solar, wind, nuclear, fuel cell systems, Battery Energy Storage Systems (BESS), and Flywheel Energy Storage Systems (FESS). To encounter system performance requirements, the PID and FOPID controller parameters are adjusted using a metaheuristic algorithm, namely Artificial-Bee-Colony (ABC) optimization algorithm. Metaheuristic optimization algorithms always do not guarantee global maxima/minima. Hence, another metaheuristic optimization algorithm, Teaching-Learning-based Optimization (TLBO), is used to validate the results. The results clearly show that the optimal PID and FOPID controllers can handle the system frequency and maintain the stability of the studied N-R HES.

## 1 Introduction

Electricity has a significant consequence on modern civilization. With the increasing population, the energy demand is growing. Thus, humans must shift towards alternative sustainable energy sources. Nuclear and renewable energy sources are the most convenient solutions for reducing greenhouse gas emissions and the usage of fossil fuels. Out of all renewable energy sources, solar and wind energy are commonly used due to their availability, easy installment, and risk-free operation. Nuclear energy is an excellent alternative to fossil fuel energy due to its' capacity factor. Therefore, power generation through the hybrid energy system, which is the integration of nuclear and renewable energy, should be encouraged.

The vicinity around Nuclear Power Plants (NPPs) is risky, so it is kept nearly uninhabited to reduce exposure. This no-man zone, named an exclusion zone, can be utilized by erecting sustainable energy sources, either sun-based or wind-based, if barometrical conditions are adequate. A renewable energy system can be used in the exclusion zone to make the nuclear power plant more profitable if atmospheric factors, such as wind speed and solar irradiance, are suitable [1].

Gabbar et al. attempted to incorporate renewable energy with nuclear energy to maximize the use of existing energy infrastructure or meet local energy requirements, especially in faraway locations [2]. The authors conducted the feasibility analysis of nuclear-renewable integrated energy systems by HOMER (Hybrid Optimization Models for Energy Resources) software. The results of their HOMER simulation suggested that nuclear-renewable hybrid energy systems could be the most viable option for meeting electricity demand in the coming days. The optimization results revealed that Nuclear-Renewable Hybrid Energy Systems (N-R HESs) can service a remote area with utmost reliability [3].

One of the evident barriers to developing and least-developed countries is greenhouse gas (GHG) emissions, which must be reduced substantially. Nuclear-renewable integrated systems excel at optimal energy distribution to various industrial schemes to reduce carbon emissions and expand profit. Though prior studies on large/small-scale nuclear-renewable integrated systems have used physical component-based simulators like Modelica and RAVEN, MATLAB simulator is being used nowadays. Few studies have already been done regarding N-R HES hybridization approaches of mathematical modeling and comparison in MATLAB simulator. Particle Swarm Optimization (PSO), a metaheuristic optimization technique, has also been developed and implemented in the suggested system layout to determine the optimal N-R HES configurations [2].

Bragg-Sitton et al. listed several benefits of combined Nuclear-Renewable energy systems, such as carbon emissions will be minimized in N-R systems, and global warming will be addressed. N-R systems assist in making renewable energy more competitive and extend green energy for future generations. N-R infrastructures also enable grid-scale energy storage for modernizing the grid infrastructure [4].

A complete mathematical model of a nuclear reactor is required to investigate the load-following capabilities of reactors. Also, nuclear reactors must be compatible with the medium and long-term stability study of power systems. The nuclear power generating unit typically changes its' output power to a certain extent, but the power ramp is not abrupt. As a result, the nuclear power plant usually acts as a baseload plant. Nuclear power generating units are seldom affected by grid voltage fluctuations at baseload operation [5].

Nuclear reactors are nonlinear by nature, and their characteristics change over time due to their power output. However, given the growing role of power plants in energy generation, load-following nuclear reactor operation may appear to be unavoidable in the future. Therefore, the inclusion of PID controllers in nuclear reactors would be a complex task. Hence, Liu et al. studied combining a fuzzy logic controller and a proportional-integral-derivative (PID) to form a fuzzy proportional-integral-derivative (fuzzy-PID) and proved its outstanding performance. The method appeared to be relatively easy and practical for complicated and nonlinear nuclear power reactor control systems, based on the process of creating and optimizing Fuzzy-PID [6].

Regad et al. studied an autonomous microgrid system consisting of wind, solar, diesel engine generator, fuel cells system, battery system, flywheel energy storage system, and a proportional-integrator-derivative (PID) controller for frequency regulation. Because of the stochastic nature of solar and wind, frequency and power fluctuate frequently. PID controller is one of the power system components that can perfectly control the frequency [7].

Solar Photovoltaic (SPV) and Wind Turbine Generator (WTG) RESs were combined with traditional thermal and hydro stations in a typical hybrid power system-studied by Datta et al. During abundant generation from RESs, water heaters were employed to utilize the excess generation. This microgrid was subjected to sudden changes in load demands or renewable energy generation. Therefore, keeping the system frequency as accurate as possible for regular operation is quite challenging in this type of system [8].

Optimal tuning of controllers is another vital issue encountered in complex energy infrastructures. Izci et al. showed a Fractional Order PID (FOPID) control system was utilized to regulate a DC motor's speed and maintain terminal voltage levels in automatic voltage regulators. An opposition-based Hybrid Slime Mold with a Simulated Annealing (SA) algorithm was used here for tuning the parameters of the FOPID controller. The proposed algorithm improved the original Slime Mold Algorithm (SMA) by using opposition-based learning for a better exploration and simulated annealing for exploitation [9]. Izci et al. also proposed an augmented hunger game search algorithm using a learning technique based on logarithmic spiral opposition. The authors proved that LsOBL-HGS could efficiently design control systems and optimize functions [10].

Finding a controller that is both convenient and designed with the critical approach is a considerable challenge for power converters. Controllers can be linear and nonlinear. Nonlinear controllers can be more dynamic but are also complex. Therefore, linear converters are often more desirable. FOPID controllers are handy as they are a version of PID controllers, a commonly used linear controller, and are more capable of performance optimization. Power converters are nonlinear systems and require highly efficient control systems [11]. Izci et al. proposed a FOPID controller to deal with such a challenge. A new metaheuristic algorithm is also developed for tuning the FOPID controller to optimize a buck converter system [12]. The authors also developed an original hybrid metaheuristic algorithm to optimize an automobile cruise control system. The design included a Bode’s ideal transfer function-based PID controller. The algorithm, named AOA-NM, uses Arithmetic Optimized Algorithm (AOA) for exploration combined with the Nelder-Mead (NM) simplex search for completing tasks that are exploitive in nature [13]. Furthermore, the authors covered the creation of a novel-enhanced metaheuristic algorithm employing the HGS algorithm and a modified OBL scheme in another study. A FOPID controller used in a magnetic ball suspension system has been suggested as a candidate. A newly created unique algorithm named modified opposition-based hunger games search (mOBL-HGS) has been introduced here to tune the FOPID controller effectively. Extensive analyses using the CEC2017 test suite have demonstrated that the mOBL-HGS algorithm performed better than the GWO, HHO, AO, and original form of the HGS algorithm [14].

In the presence of renewable energy sources and storage devices, optimal FOPID controllers can be helpful in microgrids to limit the influence of frequency and power fluctuations. FOPID controllers can also be used to increase the performance of the microgrid system and can be tuned using metaheuristic optimization algorithms, such as Genetic Algorithm [15]. Interconnected microgrids can deliver clean and sustainable electricity during normal and emergency operations. Microgrids reduce dependency on the electric grid while also providing a flexible and adaptable energy supply. Othman et al. utilized the Enhanced Nature-Inspired Meta-Heuristic (ENIMH) optimization algorithm, a metaheuristic optimization algorithm, to tune PID controllers in a microgrid system and investigate the performance of the microgrid system. The algorithm demonstrates excellent capability in achieving the optimum solution by identifying and exploiting the best result. The proposed approach considerably improves the dynamic responses of the microgrid [16].

Due to the intermittent nature of wind and solar electricity, frequency variation is a severe issue in the hybrid energy system. Therefore, incorporating new techniques with PID controller are encouraged nowadays to minimize the frequency deviation. One of those techniques is the PID controller with filtering (PIDF) technique. Compared to conventional PI controllers, this new control system is superior in terms of settling time, percentage of overshoots and undershoots, and oscillation. The PIDF controller is also found to perform well in the research when the system is subjected to random load variation and unexpected wind input [17].

Yang et al. proposed an original FOPID controller based on a perturbation observer (PoFOPID) controller for obtaining the highest possible amount of solar energy. The authors used photovoltaic cells connected to electric grids in different weather conditions. Using FOPID controllers allows for compensation of the perturbation estimate and thus drastically increases dynamics. A Yin Yang pair optimization algorithm was used for quickly tuning the controller’s parameters. The PoFOPID combines the consistency and reliability of a control system based on a perturbation observer with the global optimality of the Yin Yang Pair algorithm [18].

Yang et al. also demonstrated the control of a wind energy conversion system based on a permanent magnet synchronous generator (PMSG). An innovative adaptive fractional-order PID (AFOPID) controller was used here, which maximizes energy conversion by utilizing a linear perturbation observer. The parameter was tuned using the Particle Swarm Optimization (PSO) technique [19].

Metaheuristic optimization algorithms can also be implemented in different fields of study. Izci et al. presented a meta-heuristic Arithmetic Optimization Algorithm (AOA) for application in biomedicine. The algorithm has been built using the greedy selection from different evolution algorithms and the Logarithmic spiral (Ls) search from the whale optimization algorithm. It has been benchmarked using unimodal and multimodal benchmark functions. The algorithm is more efficient than other metaheuristic algorithms found in the literature. Later, Ls-AOA was used in this research to design a PID controller employed in a Functional Electrical System (FES). Statistical tests and convergence profiles demonstrated an improvement in the PID performance. Sine-cosine, particle swarm algorithm, original arithmetic optimization Algorithm, and the conventionally used Ziegler-Nichols tuning scheme were then used for comparative analysis of the frequency and transient response of this FES system. Besides, their noise elimination and disturbance rejection capability were also evaluated. These assessments showed that the Ls-AOA algorithm was more capable of use in FES systems [20].

This paper deals with two classic controllers to evaluate the microgrid system's control performance, primarily simulated by a nuclear reactor, renewable energy systems, and energy storage. The research intends to develop PID and FOPID controller-based nuclear-renewable hybrid energy systems for frequency regulation. The optimal tuning of the PID and FOPID controllers is conducted by two meta-heuristic algorithms, namely the Artificial Bee Colony algorithm (ABC) and Teaching-learning-based optimization (TLBO). It is crucial to obtain the appropriate gain parameters of a controller and accomplish the specified objective. Therefore, ABC and TLBO optimization techniques are studied here. The ABC and TLBO algorithms are effective and quick converging optimization techniques for resolving global optimization issues. The model of the microgrid system has been implemented in MATLAB/SIMULINK.

## 2 System modeling

A microgrid is a system of interconnected distributed power production, including loads and resources. As a result, it can perform whether connected to load or disconnected from the electric power grid. Usually, energy sources, power converters, efficient transformers, and storage devices make up microgrid systems.

In this research, the microgrid systems consist of solar, wind, nuclear, fuel cell systems, and energy storage systems, i.e., Battery Energy Storage Systems (BESS) and flywheel energy storage systems (FESS). Large deterministic drift and small stochastic power fluctuations are considered for wind generation, solar generation, and electric load. The fluctuations are modeled in a general template. This type of framework produces a time series with minor stochastic variations around the mean or demand power. Furthermore, the models provide for a quick shift in the mean value to mimic real-world events in which these parameters vary significantly. Because of the unexpected change in base value along with stochastic fluctuations, the origin of such unknown behavior in the power generation and demand can be designed in the same template, having different parameters.

The power profiles obtained from renewable energy systems, such as solar and wind, are integrated first into this research. Then, the remaining energy systems, such as nuclear, fuel cell systems, and flywheel energy storage systems, are blended. The fuel cell will take the response from the microgrid and decide whether to store the energy or provide it to the network. If power is needed, the microgrid will take energy from the battery energy storage systems. In this integrated system, the error property has been balanced by two types of controllers- PID controller and FOPID controller.

The small stochastic power fluctuation and large deterministic drift calculating for solar power, wind power, and electric demand can be written as follows [21].

(1) $$P=\left(\varphi .ɳ.\sqrt{\beta \frac{\left(1-G\left(s\right)+\beta \right)}{\beta }}\right)\Gamma =\chi .\Gamma$$

where φ is the stochastic component of the power, P represents the wind or solar and load powers, β presents the mean value of the power, ɳ is a constant that normalizes the generated power or demand powers, χ is a constant corresponding to the per unit (p.u.), and Γ is time-dependent switching signal with a gain causes the fluctuation of the value for stochastic powers. It is a time-dependent switching signal with a gain that dictates the sudden fluctuation in the mean value for the stochastic power output. G(s) is a low pass filter.

### 2.1 Solar energy

Renewable energy is emerging as a viable source of clean energy resources. Recent advancements in smart microgrids have made these systems a viable option for decentralizing power generation, increasing operational efficiency, and ensuring reliability. And Photovoltaic (PV) is one of the most prevalent renewable energy sources [22].

A PV system is a type of energy source that converts solar radiation into electrical energy. The photo effect transfers the radiation energy straight to the electrons in their PV crystals. Solar energy has long been regarded as a primary source of non-polluting energy. The unpredictability of solar energy is a typical disadvantage. For a significant period of the year, standalone PV arrays do not produce valuable electricity. In general, the changes in solar and wind energy do not correspond to the demand distribution throughout the time [23]. For the solar power generation, the parameters of Eq. (1) are listed below. Eqs. (2) and (3) represents the corresponding values from Eq. (1) for solar energy [21].

Φ∼U(−1,1),ɳ=0.9,β=10

(2) $$G\left(s\right)=\frac{1}{10^{4}.s+1}$$

(3) Γ=0.05h(t)−0.02h(t−180)

### 2.2 Wind energy

Wind energy systems are among the most secure and reliable renewable energy sources, and they are widely employed. The generating units have gained recognition because of their environmentally favorable properties, limitless energy sources, and rapid technological advancements. According to the nations' development and environmental pollution, the usage of this source has recently expanded enormously [15].

Under power supply failures and variations in wind speed, Wind Turbine (WT) power output alters. When the wind speed varies, the output power of WTs changes with a small-time delay due to the inertia of WTs. In contrast to the classic component-based equivalent modeling method, the transfer function modeling technique sees the identical model as an input/output model with no clear physical relevance. The accuracy of the model created using the component-based equivalent modeling approach is higher than that of the transfer function based equivalent modeling method since WTs parameters are not necessary during the transfer function based equivalent modeling [24].

For the wind system, the parameters of Eq. (1) are presented below. Eqs. (4) and (5) represents the corresponding values from Eq. (1) for wind energy [21].

Φ∼U(−1,1),ɳ=0.8,β=10

(4) $$G\left(s\right)=\frac{1}{10^{4}.s+1}$$

(5) Γ=0.24h(t)−0.04h(t−140)

Here, h(t) is the Heaviside function.

For the demand load, the parameters of Eq. (1) are written as follows. Eqs. (6) and (7) represents the corresponding values from Eq. (1) for demand load [21].

Φ∼U(−1,1),ɳ=0.9,β=10

(6) $$G\left(s\right)=\frac{300}{300s+1}+\frac{1}{1800s+1}$$

(7) $$\Gamma =\frac{1}{\chi }+\left[\begin{array}{c} 0.09h\left(t\right)+0.03h\left(t-110\right)\\ +0.03h\left(t-130\right)+0.03h\left(t-150\right)\\ -0.15h\left(t-170\right)+0.1h\left(t-190\right) \end{array}\right]+0.02h\left(t\right)$$

### 2.3 Nuclear energy

The formulation of a nuclear reactor's transfer function is a fundamental feature of this research. It explains how a change in the neutron flux at a specific location and time affects the entire system. The determination of typical reactor characteristics is attainable in the event of a known perturbation [25].

Transfer function of the governor of a Nuclear Power Plant (NPP) is given in Eq. (8) [1].

(8) $$\frac{Pm}{Pv}=\frac{1}{0.08s+1}$$

Transfer Function of High-Pressure Turbine, Low-Pressure Turbine_1, Low-Pressure Turbine_2, Reheater_1, and Reheater_2 can be described as follows by Eqs. (9), (10), (11), (12), and (13), respectively [1].

(9) $$\frac{Fn}{Pv}=\frac{2}{0.5s+1}$$

(10) $$T_{LP1}\left(s\right)=\frac{0.3}{0.05s+1}$$

(11) $$T_{LP2}\left(s\right)=\frac{5s+1}{10s+1}$$

(12) $$T_{RH1}\left(s\right)=\frac{1}{7s+1}$$

(13) $$T_{RH2}\left(s\right)=\frac{1}{9s+1}$$

### 2.4 Flywheel energy storage system (FESS)

An AC system’s total electrical energy cannot be stored electronically. Instead, electric energy can be stored in the form of electromagnetic, electrochemical, kinetic, and potential energy within a microgrid. Two characteristics distinguish energy storage technology applications: firstly, the amount of energy that the component can store; secondly, the energy transfer rate into and out of the storage device [26].

A FESS consists of a reversible motor/generator unit, bearings group, power electronics system, and a vacuum chamber. The generated energy is transferred to or from the flywheel system completed by a motor/generator unit in the FESS, which employs electrical power for acceleration and deceleration. The amount of energy stored in the system is determined by the flywheel's angular velocity and moment of inertia. The apparatus is put inside a vacuum to prevent strong winds and power losses [27].

### 2.5 Battery energy storage system (BESS)

Due to the fickle nature of renewable energy sources, there are significant problems, such as energy generation and load maintenance, for the system’s stability and reliability. Many practical solutions have been aimed to meet these needs, including Electrical Energy Storage (EES)-battery bank. EES is a type of energy storage that allows for load shifting through demand management and grid connectivity.

Electrochemical cells are coupled in series or parallel in a BESS to generate electricity at a desired voltage. With the help of cells, energy is converted bi-directionally- between electrical and chemical energy. Batteries can be used in a wide range of energy management and transportation applications. All batteries cannot be discharged fully because they rely on the Depth of Discharge (DoD) cycle [27].

### 2.6 Fuel cell (FC)

FCs transform chemical energy directly into electrical energy without damaging the environment or producing noise. The cells generate electricity and water using hydrogen and oxygen in separate electrodes, anode, and cathode. Lower Carbon dioxide and Sulphur dioxide emissions, reasonable efficiency relative to typical thermal machines, and high-power density are the benefits of this technology.

FCs come in various shapes and sizes, depending on the electrolyte composition, operating temperature, and fuel type. Six major types of fuel cells have been explored based on their characteristics: proton exchange membrane fuel cells, alkaline fuel cells, phosphoric acid fuel cells, solid oxide fuel cells, and molten carbonate fuel cells [28].

### 2.7 Microgrid system with PID controller

A Proportional Integrator Derivative (PID) controller can balance the microgrid frequency errors and control the system frequency by error balancing. Usually, PID controllers are utilized in industrial control applications to control temperature, flow, pressure, speed, and other process variables. Since PID controllers employ a control loop feedback mechanism to regulate process variables, this type of controller is one of the most precise and reliable controllers. Closed-loop control feedback is used in a PID control to keep a process’s actual output close to the target or setpoint output. A PID controller works to control the frequency when there's a change indicated in the system. The frequency of the whole system is managed by maintaining the energy storage in this study.

However, PID can only predict the stability zone. It is indicated by trial and error and the designer’s experience. One of the most common criteria for reducing system error and determining the appropriate PID gain values for a particular system response is integral time absolute error (ITAE), studied in this paper [29]. The cost function of this work is the Integral of Time-weighted Absolute Error (ITAE). The cost is based on the absolute difference in the output signals from the process and the reference. The mistake is weighted according to when it occurred. The absolute value ensures the cost function's monotonous expansion. The cost criteria for ITAE can be written as follows by Eq. (14) [30].

(14) $$ITAE=\int_{0}^{\infty }t\left|e\left(t\right)\right|dt=\int_{0}^{\infty }t\left|y_{r}\left(t\right)-y\left(t\right)\right|dt$$

where t is the time period, and e(t) is the error signal. There may be other cost criteria that are similar as well. It could be used to measure errors using metrics like Integral of Absolute Error (IAE), Integral of Square Error (ISE), and Integral of Time-weighted Square Error (ITSE). The use of control signals must frequently be considered when creating controllers for existing systems. Therefore, the cost function should include both error and control signals [30].

A penalty function is also introduced in the simulation of this research. When the fitness function violates the constraints specified in the simulation, the penalty function is added to the fitness function. It makes the ITAE value greater and helps to sort out the lowest ITAE value with optimal controller parameters.

The research is conducted in MATLAB/SIMULINK. For designing a PID controller in this platform by using the ITAE performance index, some necessary measures have to be taken, listed as follows.

i) In Simulink, a process model is created that includes the controller algorithms.
ii) A MATLAB m-file is built to calculate the ITAE index using a fitness function.
iii) To reduce the ITAE index, MATLAB Optimization Toolbox tool is used.

**Figure 1:**
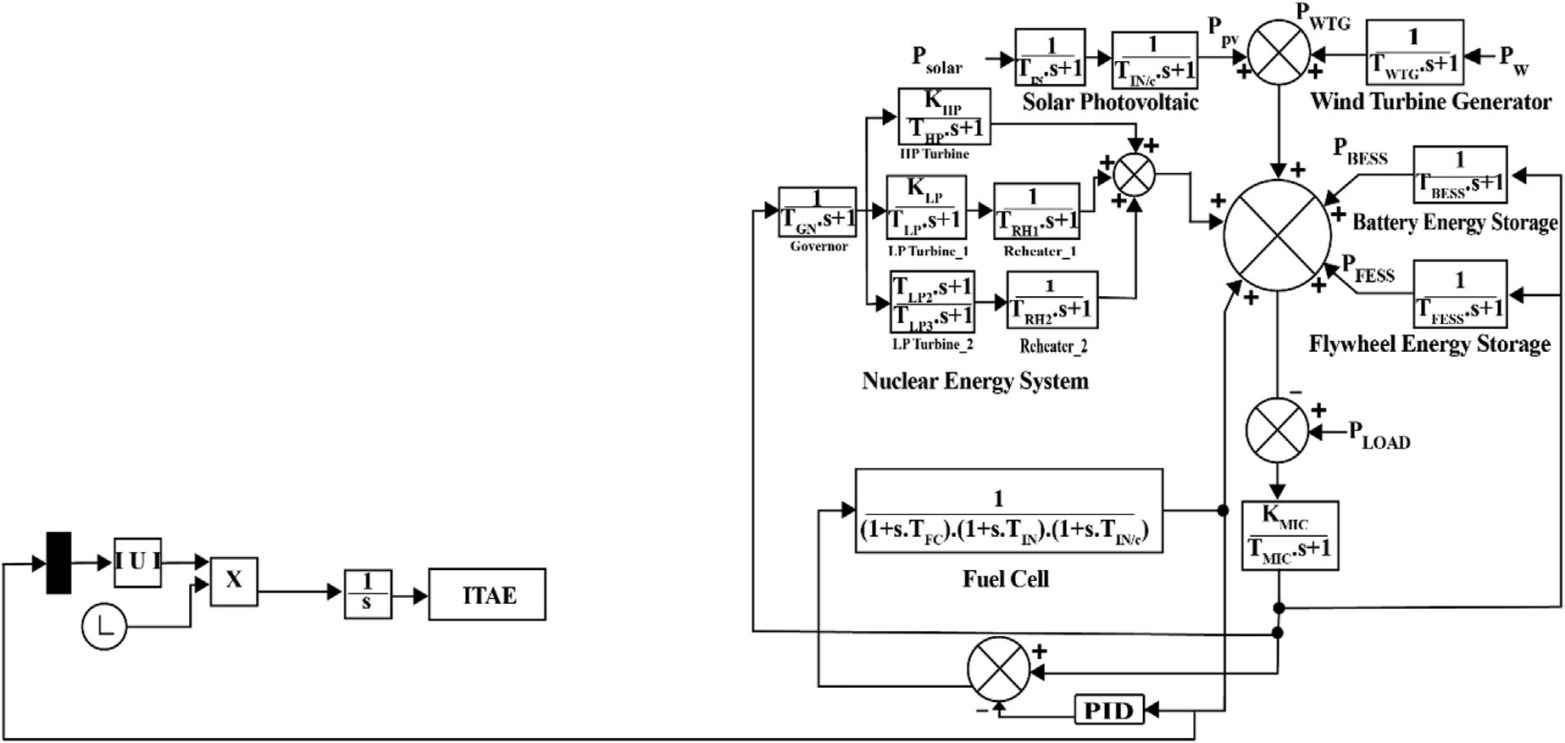
Microgrid system with PID controller.

The schematic of the microgrid with PID controller is presented in Figure 1.

### 2.8 Microgrid system with FOPID controller

The conventional PID controller is upgraded to a controller named the FOPID controller, which is based on fractional-order calculus. The FOPID controller surpasses the standard PID controller in terms of system stability. The insertion of fractional order terms λ and μ has increased effectiveness. Even though the FOPID controller has several improvements over a traditional PID controller, the additional terms in the FOPID controller make tuning more complex.

Fractional-order controllers can offer the control system more flexible time and frequency responses. It only occurs if the fractional-order controller has more degrees of freedom (DOF) than its integer-order counterpart. For instance, utilizing a conventional integer-order PID controller, which only has three parameters that can be tuned, will only allow to satisfy up to three criteria of choice. The performance of the overall control system may improve with the application of FOPID. This is because, in contrast to the typical classical PID controller, having three parameters to be tuned for three robustness criteria, employing a FOPID might meet up to five robustness requirements. FOPID is preferable because it can adjust five parameters: KP, KI, KD, μ, and λ if four or five robustness criterion is required. A FOPID controller can satisfy up to five robustness criteria: phase margin specification, gain cross-over frequency specification, isodamping-robustness to gain variations, complementary sensitivity specification, sensitivity specification, and elimination of steady-state error [31].

**Figure 2:**
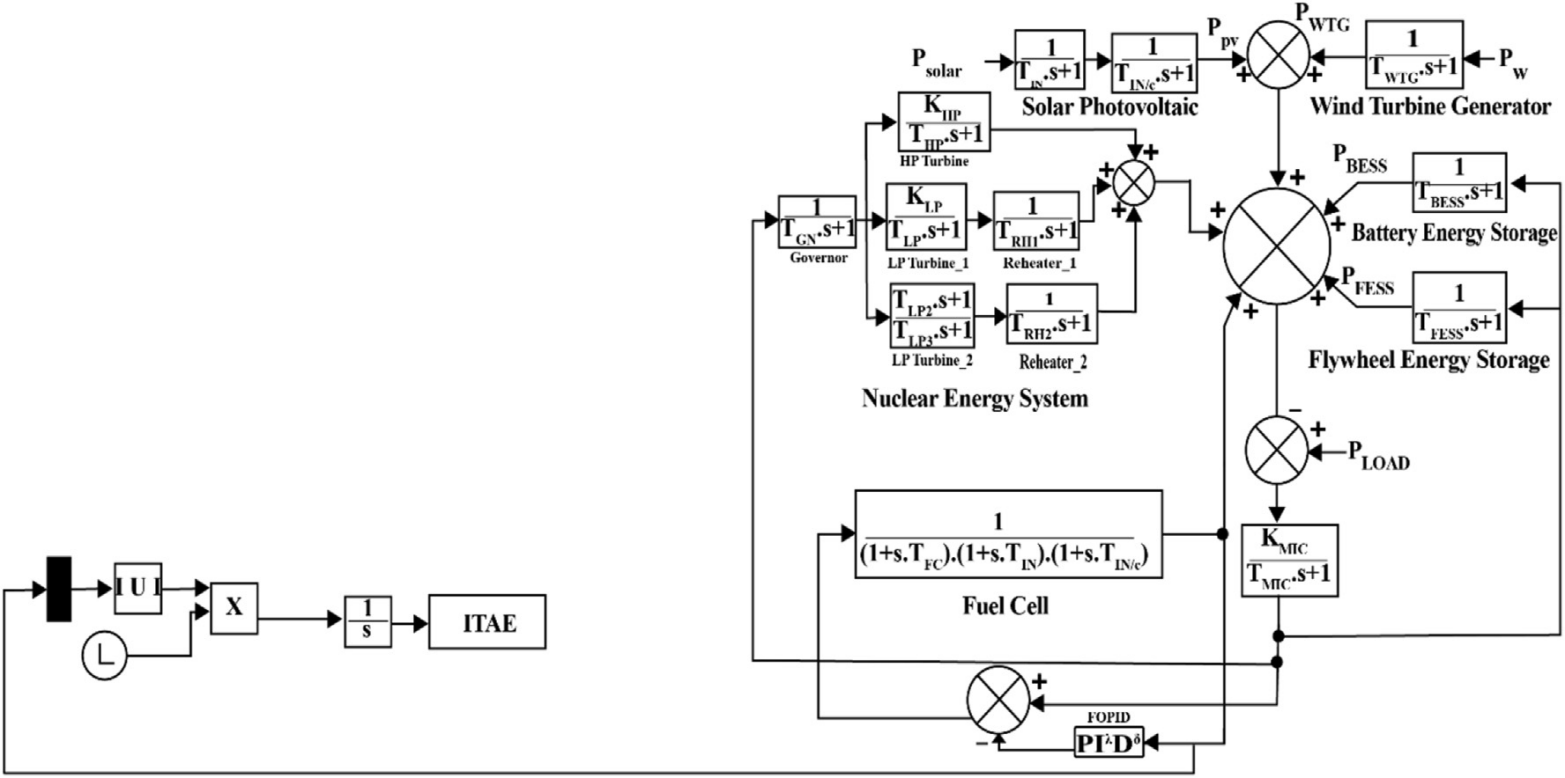
Microgrid system with FOPID controller.

The mathematical expression of a FOPID controller is presented below in Eq. (15) [32].

(15) $$G_{f}\left(s\right)=\frac{U\left(s\right)}{E\left(s\right)}=K_{P}+K_{I}s^{-\lambda }+K_{D}s^{\mu }$$

where, (λ, μ) > 0. A FOPID controller usually uses more complicated approximations and requires considerable computational resources. Since PID and FOPID controllers can maintain system stability, both controllers are studied in this research. PID and FOPID controllers work instantly to regain the system’s stability if any changes occur [4]. The schematic of the microgrid system with FOPID controller is shown in Figure 2.

**Figure 3:**
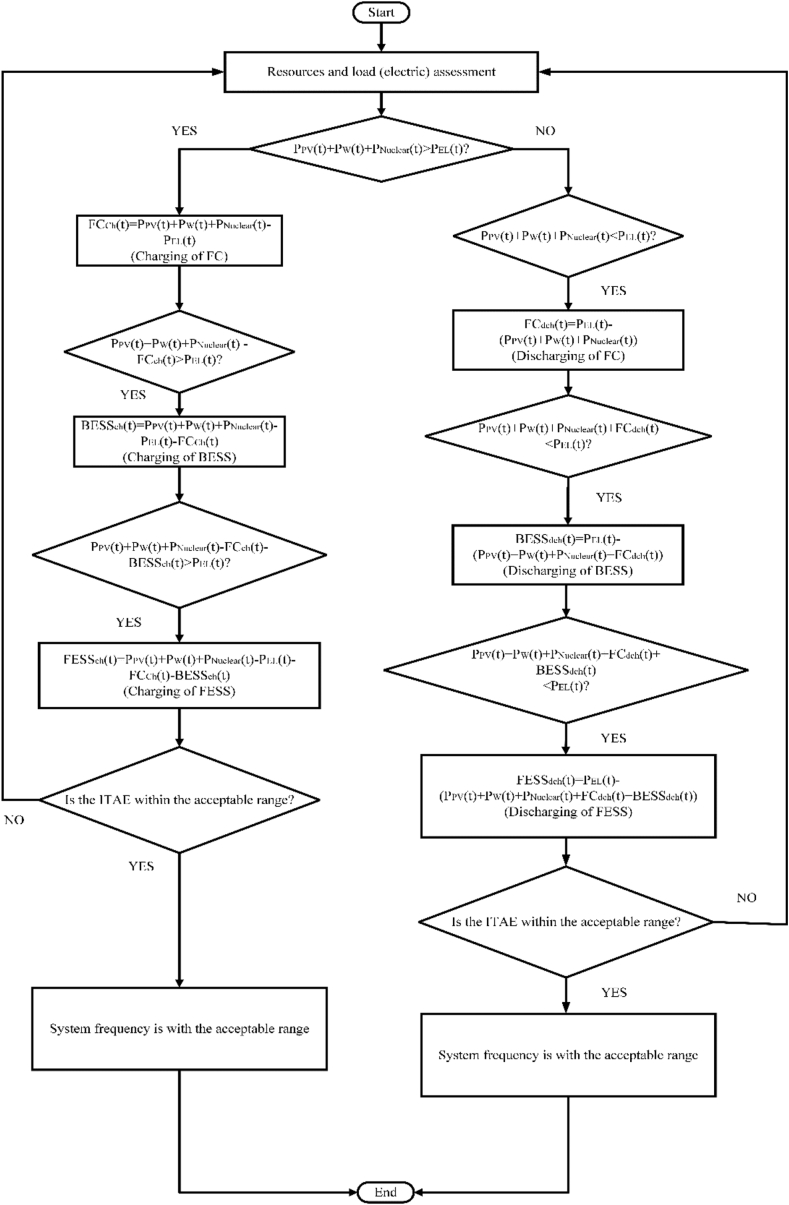
Flowchart for energy management and control strategy of the N-R HES

The energy management and control strategy of the N-R integrated microgrid system is illustrated in Figure 3. In Figure 3, the N-R HES consists of photovoltaic cells, wind turbines, fuel cell systems, nuclear reactors, battery storage, fuel cells, and flywheel storage systems. The excess energy generated from the resources is stored in the energy storage systems and compensates for the deficit energy within the integrated system. The fuel cell storage system is the primary energy repository. The batteries and the flywheel storage system are for retaining energy after the fuel cell system’s storage capacity is filled. On the other hand, if there is any power shortage in electric demand fulfillment, fuel cell, battery energy storage system, and flywheels will be discharged respectively to meet the electricity requirement. The system frequency is monitored by calculating the ITAE value. The purpose of using controllers is to minimize the ITAE value. The less the value of ITAE, the system closes to stability. The system frequency is controlled if the ITAE is within the acceptable range.

## 3 Optimization/tuning of the microgrid controllers

Optimization can be defined as determining a solution to a problem in which a single set of objective functions must be maximized or minimized within a domain containing acceptable values of variables while certain constraints must be met. Many data sets in the domain could maximize or minimize the objective function(s) while adhering to the limitations. They are referred to as acceptable solutions, and the best among them is the optimum solution to the problem. Control factors such as population size, number of generations, select the size, and so on are required for all evolutionary and swarm intelligence-based optimization techniques. Aside from the standard control settings, each algorithm requires its own set of algorithm-specific parameters.

In this paper, tuning of the controllers is accomplished by metaheuristic algorithms. A metaheuristic algorithm is an iterative strategy for guiding a subordinate heuristic by intelligently mixing several notions for exploring and utilizing search space. They are motivated by observations of natural events. A Metaheuristic algorithm is a type of stochastic algorithm that employs a mix of randomization and local search. They are frequently based on observations of nature or biological processes. Genetic algorithms, particle swarm optimization, ant colony algorithms, and bees algorithms are examples of popular metaheuristic algorithms. Global optimization is usually the goal of metaheuristic algorithms. Metaheuristic algorithms cannot always find the global maxima/minima, and there is no mathematical tool to verify whether the global maxima or minima is obtained or not. Therefore, two optimization algorithms, namely Artificial Bee Colony (ABC) and Teaching–Learning-based Optimization (TLBO), are used in this study to validate the optimization results.

### 3.1 ABC optimization algorithm

The cognitive behavior of honey bee swarms in seeking a food source inspired the artificial bee colony optimization invention. The hired bees (forager bees), spectator bees (observer bees), and scouts are the three bees in the ABC algorithm. There is only one employed bee for each food source. The number of bees engaged is proportional to the number of food sources. The employed bee of a discarded food source is obliged to work as a scout, looking for new food sources at random. Employed bees in a hive transmit information with observer bees to choose a food source to forage [33].

**Figure 4:**
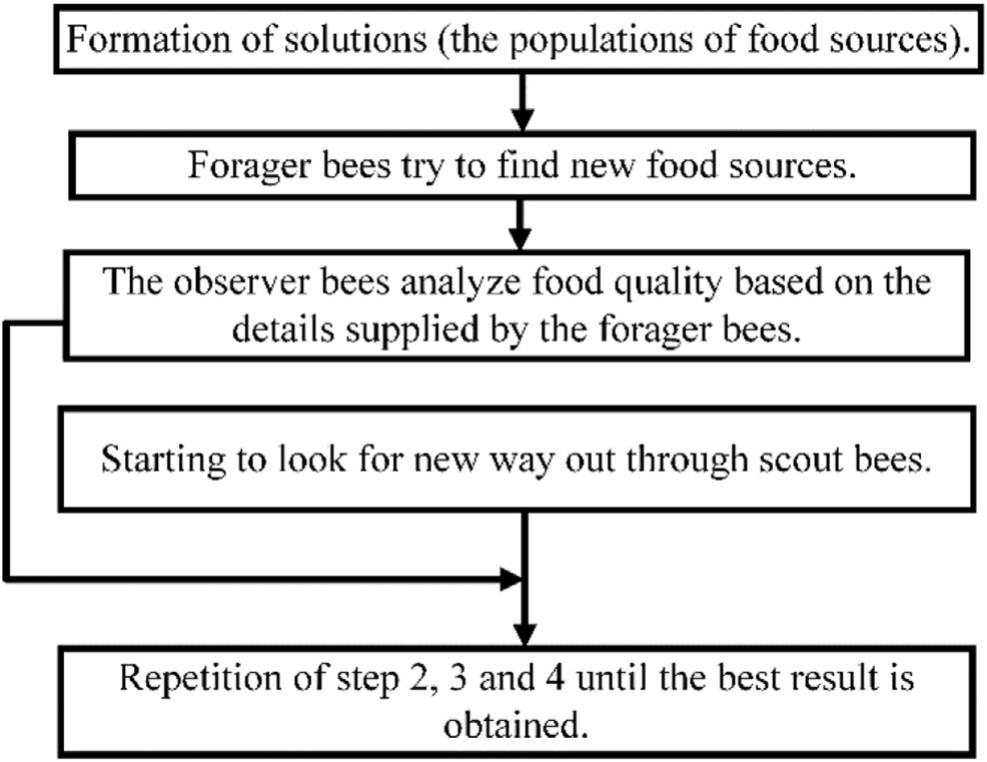
Flowchart of ABC algorithm.

The nectar amount of a food source corresponds to the quality (fitness) of the related solution in the ABC algorithm. The position of a food source reflects a feasible solution to the optimization problem. The number of employed or observer bees in the population equals the number of solutions [34]. The structure of the ABC optimization algorithm is presented in Figure 4 [35].

To indicate the amount of honey at location s, an objective function h(s) can be encoded as H(s). Thus, the probability *G*_*i*_ of an onlooker bee choosing to go to the preferred food source at ***s***_*i*_ can be defined by *G*_*i*_ = *H((****S***_*i*_*)/n)*, where *n* is the number of food sources.

The intake efficiency of a specific food source is determined by H/t, where H is the amount of nectar and t is the time spent at the food source. If a food source is attempted a particular couple of times and does not improve, it is abandoned, and the bee at that place will work in new locations at random.

Potential challenges in the ABC algorithm include combinatorial optimization, job scheduling, and web-hosting allocation [35]. An artificial observer bee selects a food source according to the probability value associated with that food source, Qi, which is computed using Eq. (16) [36].

(16) $$Q_{i}=\frac{{Fv}_{i}}{\sum_{n=1}^{N_{f}}{Fv}_{n}}$$

Here, Fv is the fitness value and $N_{f}$ is the number of food sources

The ABC optimization algorithm utilizes Eq (17) to generate a candidate food position from a recollection of an old one [36].

(17) $$U_{ab}=y_{ab}+g_{ab}\left(y_{ab}-y_{cb}\right)$$

Here, a *∈ {*1*,* 2*,..., NF}, c ∈ {*1*,* 2*,..., NF}* and *b∈ {*1*,* 2*,..., D}* are randomly selected indexes. Even though a, b, c is chosen at random, it must be distinct from each other. g_a,b_ is a random number between [−1, 1]. It controls the production of nearby food sources around y_a,b_ and displays a bee visually comparing two food positions. In this equation, *y* is the population size of the solution and U_ab_ is the produced new solution. ABC assumes that the food source has been abandoned if a position cannot be improved after a predefined number of cycles. The value of a specified number of cycles, known as the “limit” for abandonment, is an important control parameter of ABC algorithm. The scouts replace the food supply where the bees abandoned the nectar with a new food source. If the abandoned source is y_a_ and b{1, 2,..., D}, then the scout discovers a new food source which is to be replaced with y_a_. This operation can be defined by Eq. (18) [36].

(18) $$Y_{a}^{b}=Y_{\min}^{b}+rand\;\left(0,1\right)\left(Y_{\max}^{b}-Y_{\min}^{b}\right)$$

Following the production and evaluation of each candidate source position, U_ab_, by the artificial bee, its performance is compared to its predecessor. If the new food has an equivalent or superior nectar to the old source, the old one is remembered instead. Otherwise, the previous one is recognized. In other words, the selection action is carried out via a greedy selection mechanism [36].

### 3.2 TLBO optimization algorithm

The TLBO algorithm is a teaching-learning process-inspired algorithm. It assesses the impact of a teacher's involvement on a class's output. The algorithm demonstrates two primary ways of learning. The first is to learn from a teacher (known as the teacher phase), and another is to learn from other learners (known as the learner phase).

A population of learners is investigated in this optimization technique. Different subjects supplied to the students are managed by other design variables of the optimization method. A learner's result is equivalent to the optimization problem’s “efficiency” value. The “Teacher phase” and the “Learner phase” are the two components of the TLBO's operation. In the teacher’s phase, learners learn through the teacher. Throughout that phase, a teacher tries to improve the class's mean outcome in the subject; they instruct; based on their abilities. The second portion of the algorithm is the learner’s phase, where learners communicate to augment their knowledge. A learner connects with other learners at random to improve their understanding [37].

Value of the parameters that are taken for ABC and TLBO algorithm are listed in Table 1.

**Table 1:** Value of the Parameters taken for ABC and TLBO Algorithm.

Parameters	ABC	TLBO
Number of Individual Run	15	15
Variable Maximum	100	100
Variable Minimum	0.001	0.001
Number of Population	50	50
Number of Iteration	100	100

Assuming this, there are “m” number of topics (design variables) and “n” number of learners (population size, u = 1, 2,..., n) at any iteration v. Students' overall performance in a particular course can be represented by $M_{t}^{v}$, where “t” can be any number from 1 to m. The best learner (kbest) result may be determined by taking the learner population as a whole and determining who has the highest overall score ($X_{t,kbest}^{v}$) when taking into account all of the topics. The scenario can be expressed by Eq. (19) [37].

(19) $${Diff}_{{mean}_{t,u}}^{v}=r_{c}\left(X_{t,kbest}^{v}-T_{F}M_{t}^{v}\right)$$

where, $X_{t,kbest}^{v}$ is the best learner in topic t's result. T_F_ is the teaching factor that determines the change in mean value, and $r_{c}$ is a random number between 0 and 1. T_F_ can have a value of 1 or 2. T_F_’s value is determined at random with the same probability by Eq. (20) [37].

(20) $$T_{F}=round\left[1+rand\left(0,1\right)\left\{2,-1\right\}\right]$$

The TLBO algorithm does not include $T_{F}$ as a parameter. The value of $T_{F}$ is not specified. The algorithm uses a random number generator to determine the input value. Based on the ${Diff}_{{mean}_{t,u}}^{v}$, the existing solution is updated in the teacher phase according to Eq. (21) [37].

(21) $$X_{t,u,v}^{\prime }=X_{t,u,v}+{Diff}_{{mean}_{t,u}}^{v}$$

where, $X_{t,u,v}^{\prime }$ is the updated value of $X_{t,u,v}$.

In the second part of the algorithm, the learners increase their knowledge by interacting among themselves.

Randomly select two learners P and Q such that [37],

$$X_{total-P,v}^{\prime }\neq X_{total-Q,v}^{\prime }$$

where, $X_{total-P,v}^{\prime }$ and $X_{total-Q,v}^{\prime }$ are the updated function values of $X_{total-P,v}^{\prime }$ and $X_{total-Q,v}^{\prime }$ of P and Q, respectively, at the end of teacher phase. The conditions for teacher phase can be expressed by Eqs. (22) and (23) [37].

(22) $$X_{t,P,v}^{\prime \prime }=X_{t,P,v}^{\prime }+r_{c}\left(X_{t,P,v}^{\prime }-X_{t,Q,v}^{\prime }\right)\;if\;X_{total-P,v}^{\prime }<X_{total-Q,v\;}^{\prime }$$

(23) $$X_{t,P,v}^{\prime \prime }=X_{t,P,v}^{\prime }+r_{c}\left(X_{t,Q,v}^{\prime }-X_{t,P,v}^{\prime }\right)\;if\;X_{total-Q,v}^{\prime }<X_{total-P,v\;}^{\prime }$$

**Figure 5:**
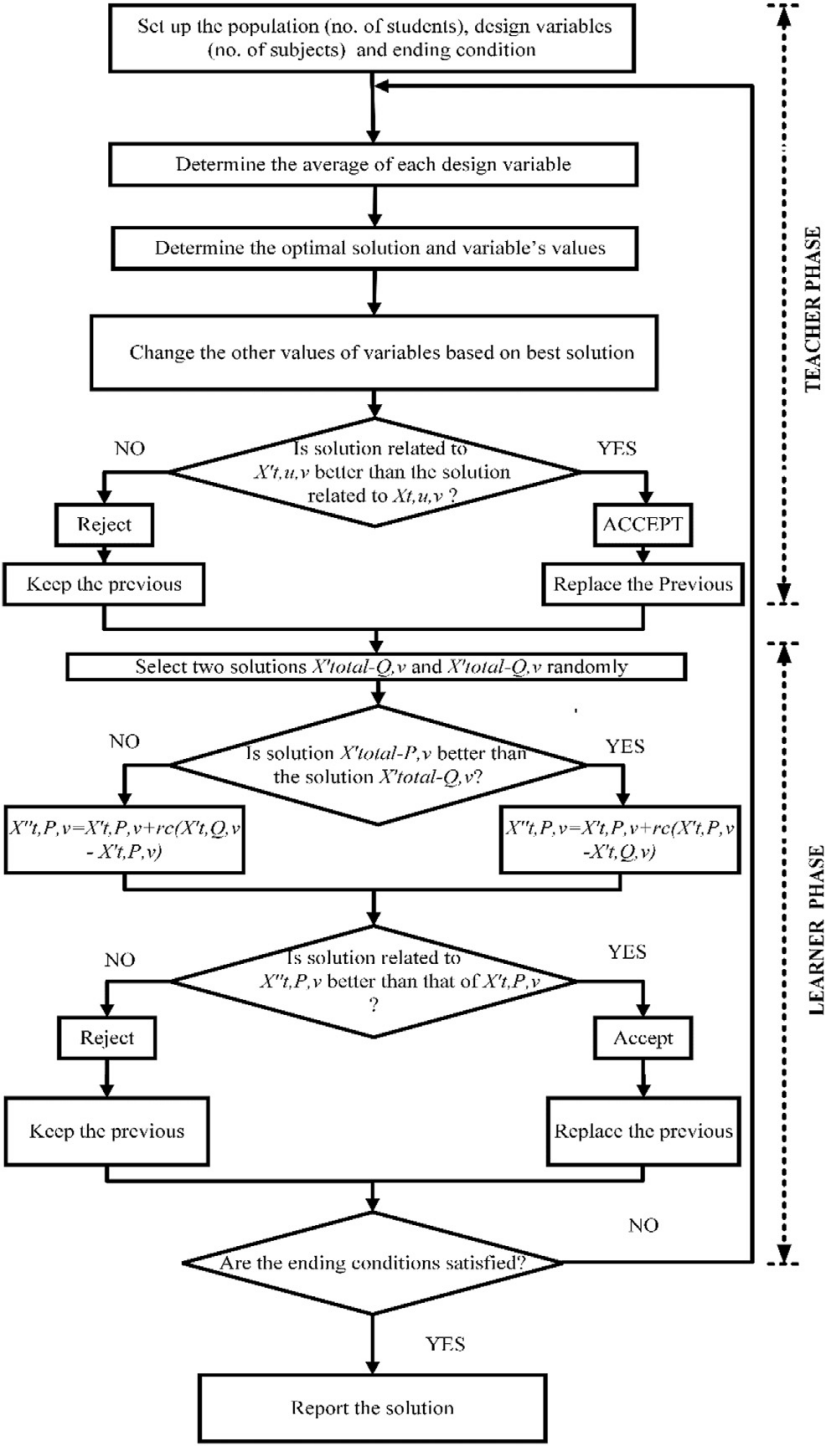
Flowchart of TLBO algorithm.

The flowchart of the TLBO algorithm is illustrated in Figure 5.

## 4 Results and simulation

In this research, it is assumed that the integrated energy system operates at a 50 Hz frequency. It usually varies from region to region throughout the world.

**Figure 6:**
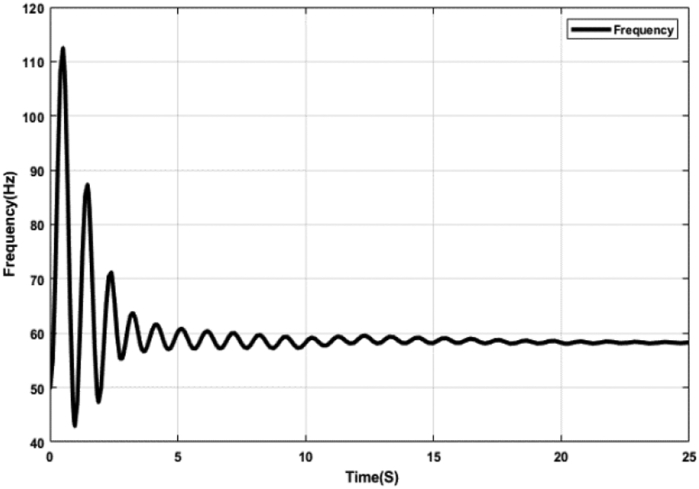
Grid Frequency without any Controller.

Without any controller, the N-R integrated energy system has less capability in maintaining a stable frequency despite the inherent controlling capacity of the hybrid system. The frequency of the N-R integrated system has exceedingly high fluctuations with respect to time and becomes stable at approximately 58 Hz, illustrated in Figure 6, which is much higher than the desired 50Hz. Though the permissible range of frequency fluctuation varies from region to region, a typical acceptable range of frequency fluctuation can be ±1.5 Hz in electric power systems [38]. But the frequency fluctuation is higher than ±1.5 Hz in this case. Thus, it demonstrates the requirement of a control system for maintaining the frequency in the N-R hybrid system at its predefined value.

In literature, the reduction of ITAE is frequently mentioned as a fitness function for obtaining optimal settings of PID and FOPID controllers. MATLAB optimization toolbox can interact easily with Simulink to implement the ITAE criterion.

**Figure 7:**
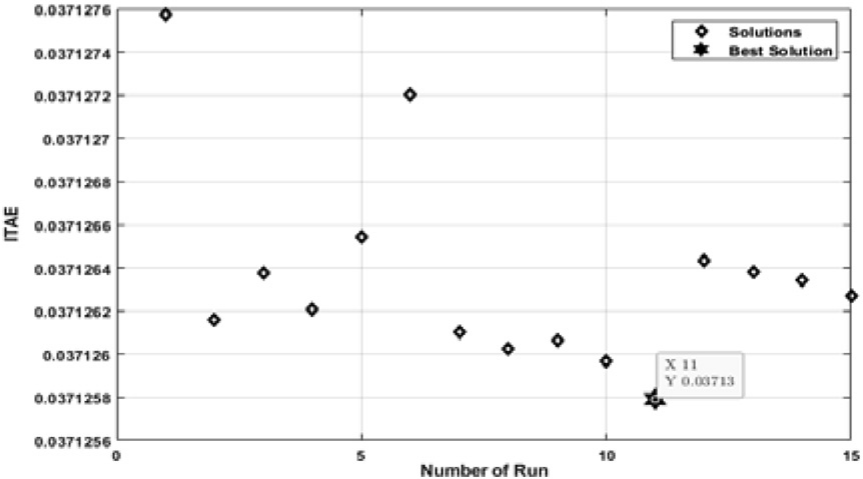
ITAE solution for each number of individual run for PID controller tuned by ABC algorithm.

**Figure 8:**
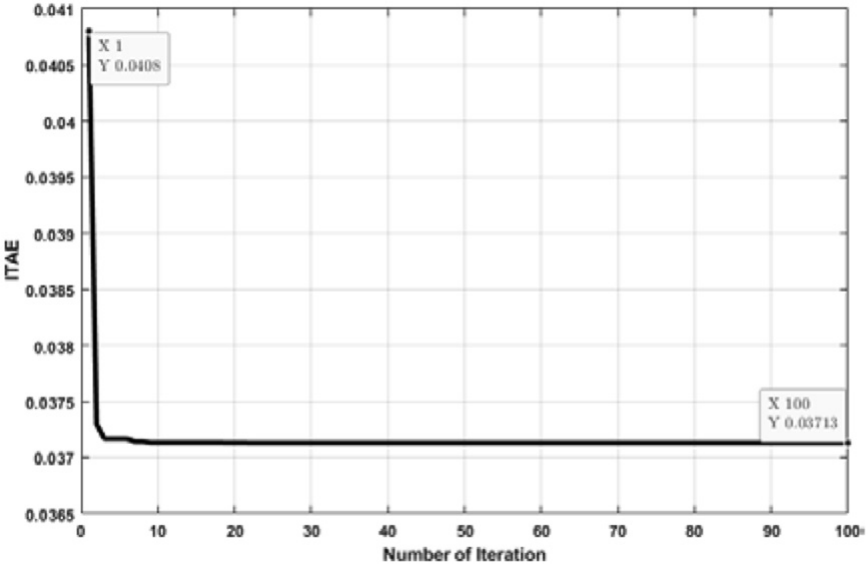
Convergence curve (PID controller tuned by ABC algorithm).

Figure 7 shows the relation between the ITAE and the number of individual runs while the ABC algorithm tunes the PID controller. Here, the ITAE solution is slightly different for each run. However, the lowest solution of ITAE is taken as the optimal solution. From Figure 7, it is observed that the optimal solution is obtained at the 11th run, and the value of ITAE is 0.03713. The optimal solution includes a hundred iterations depicted in Figure 8. From Figure 8, it is visible that the ITAE value gets minimized along with each iteration. The number of iterations is taken as hundreds (100) for all cases in this analysis, which is deemed a conservation assumption for this type of research.

**Figure 9:**
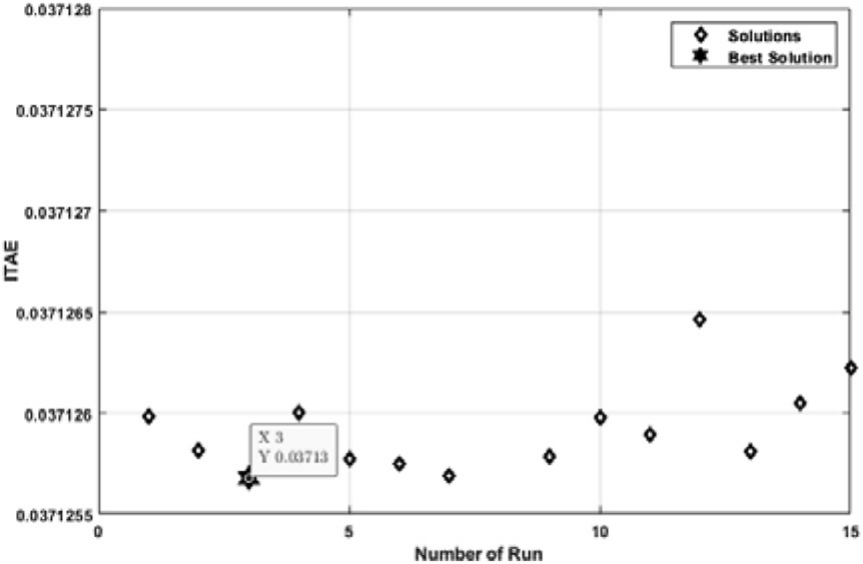
ITAE solution for each number of individual run for PID controller tuned by TLBO algorithm.

**Figure 10:**
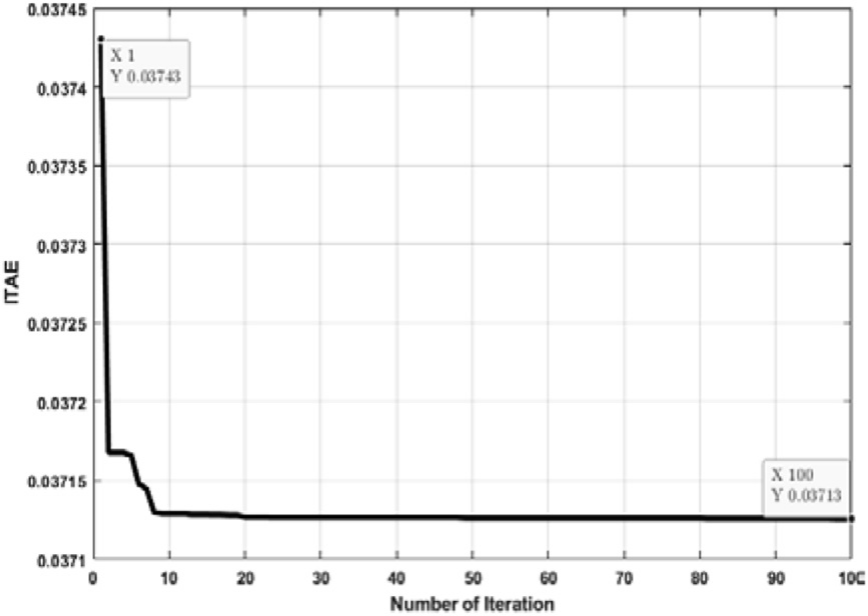
Convergence curve (PID controller tuned by TLBO algorithm).

When the TLBO algorithm adjusts the PID controller, the optimal value is obtained in the 3rd run in Figure 9. The optimal value is identical to the value obtained for the ABC algorithm. The lowest value of ITAE for PID controller tuned by TLBO algorithm is also 0.03713, which evidently validates the result obtained in Figure 7. Figure 10 illustrates the convergence of the optimal solution obtained in Figure 9.

**Figure 11:**
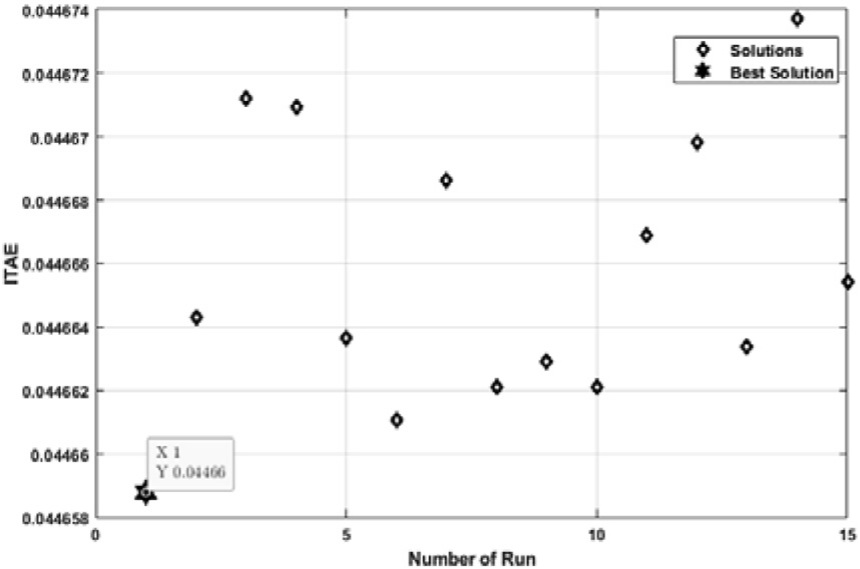
ITAE solution for each number of individual run for FOPID controller tuned by ABC algorithm.

**Figure 12:**
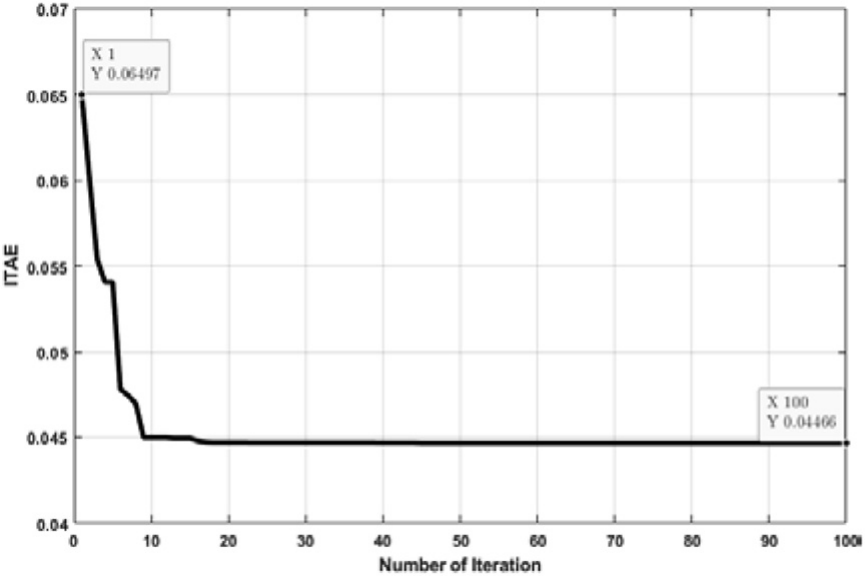
Convergence curve (FOPID controller tuned by ABC algorithm).

Figure 11 shows the relation between the ITAE and the number of individual runs for a FOPID controller tuned by the ABC algorithm. Though all the ITAE values are very close to each other, the lowest value is considered. Multiple individual runs strengthen the results in metaheuristic optimization problems. From Figure 10, it is noticed that the lowest ITAE value is obtained in 1st run, and the value of the optimal solution is 0.04466. Figure 12 shows the convergence of the ABC algorithm, similar to Figure 10.

**Figure 13:**
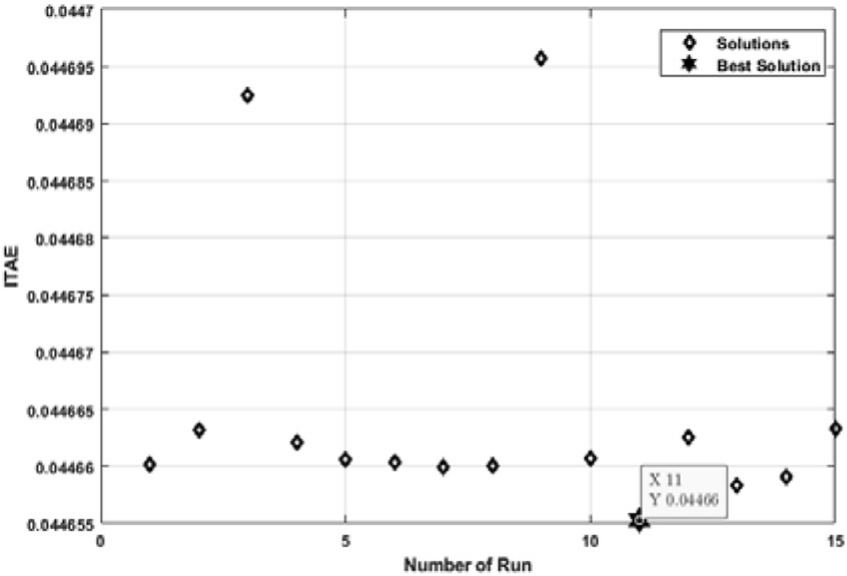
ITAE solution for each number of individual run for FOPID controller tuned by TLBO algorithm.

**Figure 14:**
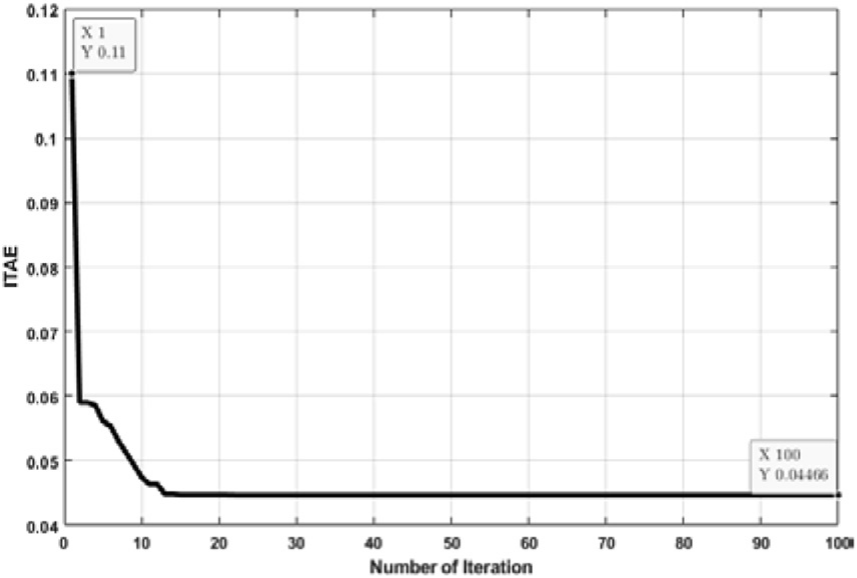
Convergence curve (FOPID controller tuned by TLBO algorithm).

Figure 13 depicts the relation between ITAE values and the number of individual runs for the FOPID controller tuned by the TLBO algorithm. The optimal value is obtained at the 11th run, and the value of optimal ITAE is 0.04466. Figure 14 represents the convergence plot of the TLBO algorithm for the FOPID controller.

Optimal values that are obtained for each parameter of the PID and FOPID controllers are recorded in Tables 2 and 3, respectively.

**Table 2:** Optimal Value for each Parameter of PID Controller.

K_p_	K_i_	K_d_
99.7926	100	0.0018

**Table 3:** Optimal Value for each Parameter of FOPID Controller.

K_p_	K_i_	K_d_	λ	μ
1.5488	98.9990	0.9515	86.8979	0.0010

**Figure 15:**
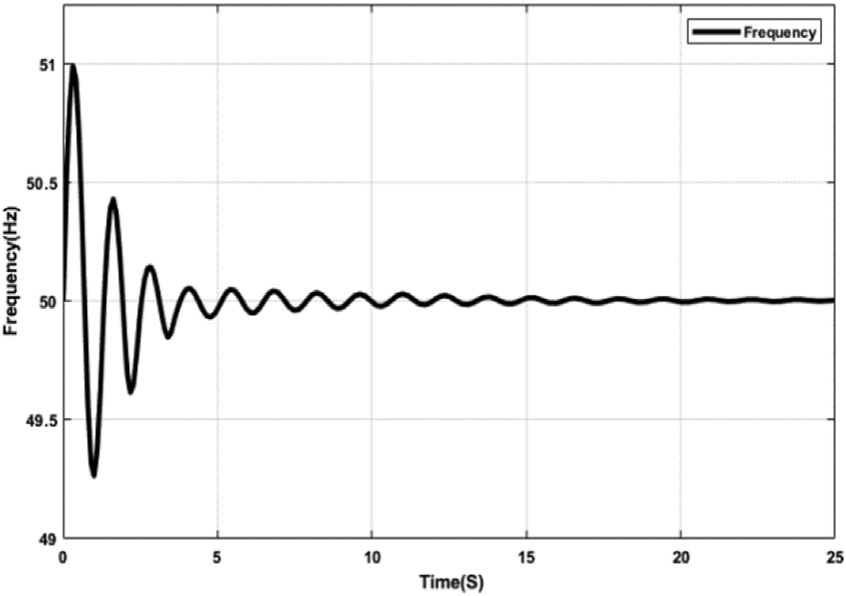
System frequency with PID controller tuned by ABC algorithm.

**Figure 16:**
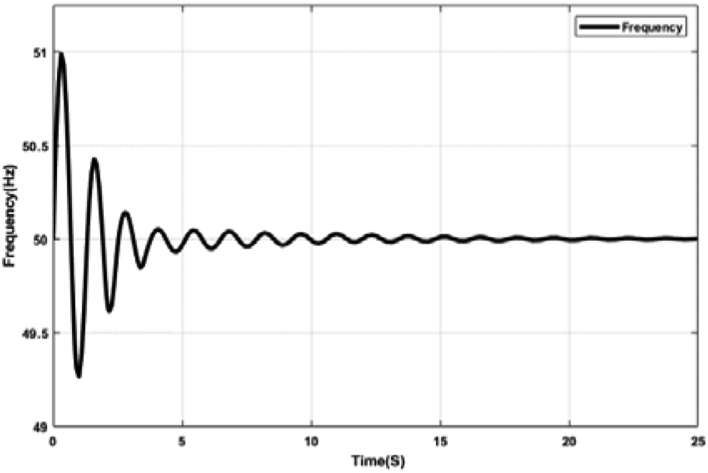
System frequency with PID controller tuned by TLBO algorithm.

Figures 15 and 16 represent the system frequency with the optimal PID controller. The diagrams demonstrate the error correction capability of the proposed optimal PID controller. The PID controller parameters have been adjusted to attain and maintain the set value at 50 Hz. Initially, as the error increases, the proportional component gradually increases the strength of the control signal to decrease the error. This action causes overshoot and raises the error again. The rapid change in frequency causes the differential controller to send a signal, which reduces this overshoot. As time passes, the integral controller also helps in maintaining the frequency at the set value. The highest deviation from the set value is 1Hz, depicted in Figures 15 and 16, which most energy systems can tolerate. PID controllers are widely used in industrial control processes because of their simple construction and excellent parameter tuning.

Table 4 summarizes the optimal ITAE solutions for the PID and FOPID controllers tuned by both algorithms. The results indicate that the least ITAE values for each controller tuned by different metaheuristic algorithms are identical, signifying the validation of optimization results.

**Table 4:** Optimal ITAE solution for different cases.

Parameters	PID Controller		FOPID Controller	
	ABC	TLBO	ABC	TLBO
ITAE Values	0.03713	0.03713	0.04466	0.04466

**Figure 17:**
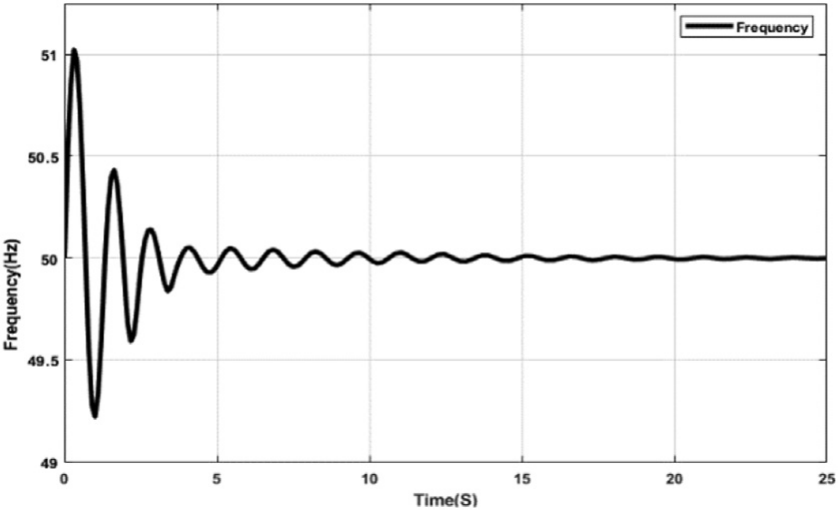
System frequency with FOPID controller tuned by ABC algorithm.

**Figure 18:**
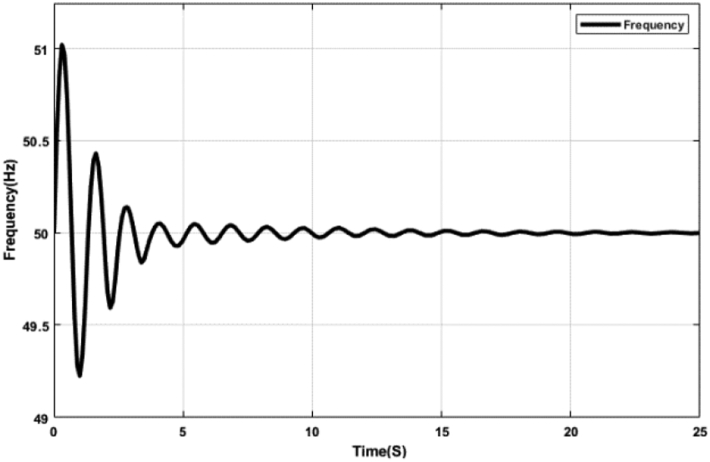
System frequency with FOPID controller tuned by TLBO algorithm.

Figures 17 and 18 represent the system frequency with the optimal FOPID controller. The proposed optimal FOPID controller can maintain system frequency at a stable condition shown in Figures 17 and 18. FOPID controller has five parameters instead of three, allowing greater flexibility and a more adjustable frequency response. But due to the higher number of parameters, the tuning of FOPID is much more complex. The tuning of the FOPID shows considerable improvement in responsiveness compared to the PID controller.

Since the integral and derivative terms were in fractional order rather than integer, FOPID controllers make control applications more robust. FOPID tuning adds two new parameters for integer and derivative order, namely λ and μ, along with the PID tuning parameters. The inclusion of two additional parameters results in a substantial improvement in process performance [39].

Table 5 shows different Key Performance Indicators (KPIs) of the system frequency graphs mentioned earlier. Rise time, settling time, and overshoot of a control system are crucial parameters, which are selected and assessed for determining the system performance. Rise time is the required time for a signal to reach from 10% to 90% of the final value. In this paper, it is calculated that the rise time for the ABC and the TLBO-tuned PID controller is 0.50 ​m ​s, while the rise time for the ABC and the TLBO-tuned FOPID controller is 0.20 ​m ​s. When there is no controller, the acquired rise time is 80.10 ​m ​s. Settling time is the time it takes for an action to stabilize. It is defined as the time it takes for a response to achieve and maintain a steady-state of 2%–5% of its ultimate value. The time it takes for the system to settle is determined by its natural frequency and responsiveness. Here, the settling time for the ABC and the TLBO-tuned PID controller is 13.03 s and for the FOPID controller is 7.65 s. The settling time with no controller is determined as 24.82 s. It is anticipated that the rise time and settling time should be less for designing a suitable control system mechanism. Furthermore, the event of a signal or function surpassing its target is known as overshoot. Overshoot occurs when a signal exceeds its steady-state value. Usually, overshoot signifies a system error. The overshoot value for the tuned PID and FOPID controllers is significantly lower than the system having no controllers, illustrated in Table 5. Table 5 indeed demonstrates the effectiveness of the optimal PID and FOPID controllers within the N-R HES.

**Table 5:** KPI of the time-domain analysis.

Characteristics	No Controller	PID	FOPID
Rise Time (ms)	80.10	0.50	0.20
Settling Time (s)	24.82	13.04	7.65
Overshoot (%)	88.24	2.04	2.19

The findings of this research are also evaluated in the frequency domain analysis. Bode plot and Nyquist diagram are used for the frequency response analysis of the studied N-R hybrid energy system. These two techniques are used to verify the result of the time-domain analysis. Since the determination of overall transfer is too complicated and time-consuming, MATLAB/Simulink is also used in this analysis.

The Bode plot should only be used to evaluate the stability of a system if it contains one or fewer phase crossover frequencies. Typically, two important parameters, gain margin and phase margin, are used in the Bode plot to determine a system’s stability. Bode plot stability criterion for a stable system is that the phase margin and gain margin should be positive, or the phase margin should be greater than the gain margin. Besides, if a system has multiple phase crossover frequencies, the lowest and highest phase crossover frequency should have sufficient gain margin for a system to be stable. An adequate gain margin between 2 dB to 10 dB is usually considered for a good closed-loop design [40].

**Figure 19:**
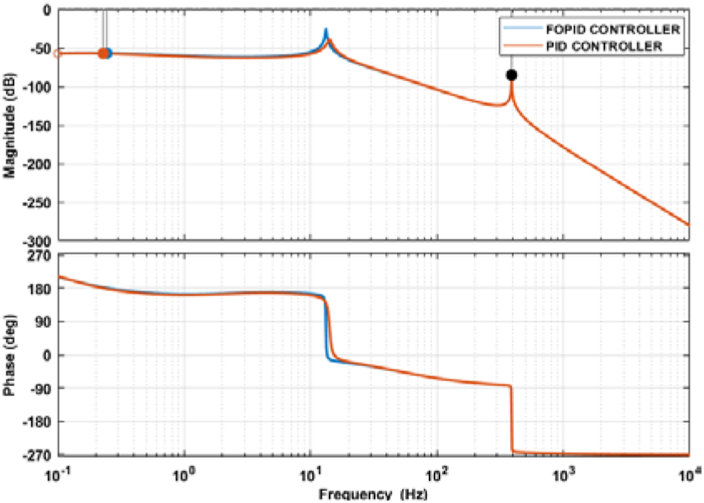
Bode plot of the PID and FOPID-controlled systems.

**Figure 20:**
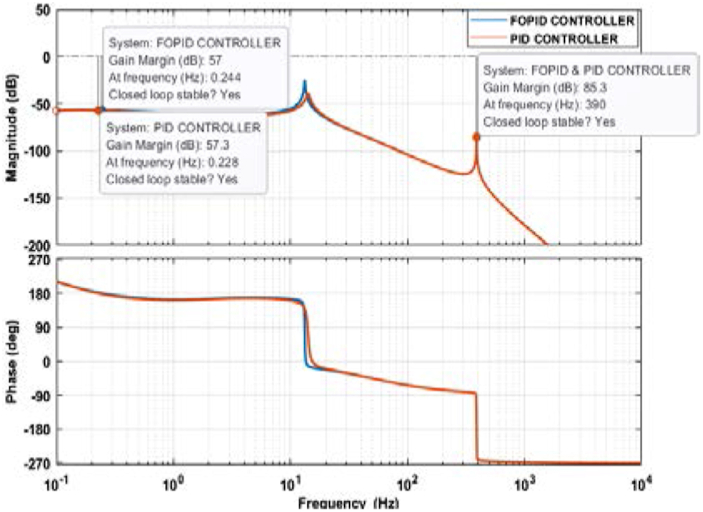
Bode plot of the PID and FOPID-controlled systems with data tip.

Figures 19 and 20 represent the Bode plot of both the PID and FOPID-controlled systems. Figure 18 shows the Bode plots without any data shown in the figure, whereas Figure 20 illustrates the graphs with the data box. For the PID-controlled system, the lowest and highest phase crossover frequencies are 0.228 Hz and 390 Hz, and the corresponding gain margins are 57.3 dB and 85.3 dB, respectively. The FOPID-controlled system has the lowest phase crossover frequency of 0.244 Hz with a gain margin of 57 Hz, while the highest phase crossover frequency is 390 Hz with a gain margin of 85.3 dB. The lowest phase crossover frequency differs for both systems, but the highest phase crossover frequency overlaps and has the same data tip in Figure 20.

The gain margin is positive, and the phase margin is infinite since the system never crosses the zero dB plane. The phase margin is positive and greater than the gain margin. Also, the multiple phase crossover frequencies have a sufficient gain margin for the systems. Thus, both the PID and FOPID-controlled systems are considered stable.

Nyquist plot is another graphical method for identifying a system's stability. From a Nyquist plot, if the critical point (−1+j0) stays outside of the encirclement, the closed-loop system can be deemed as stable. Also, if the gain margin of a Nyquist plot is greater than one, the phase margin is positive, the closed-loop system will be stable [41].

**Figure 21:**
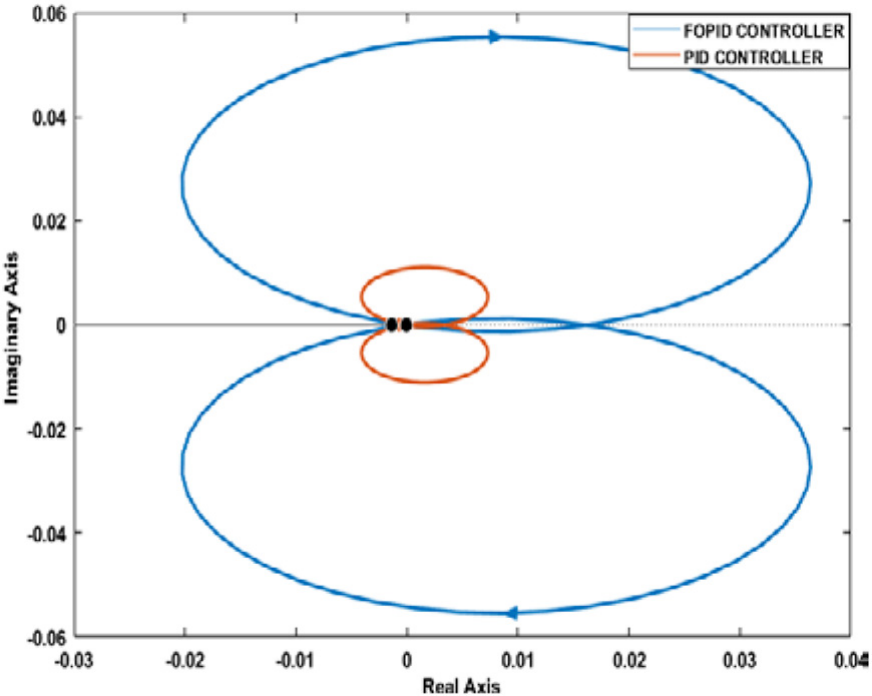
Nyquist plot of the PID and FOPID-controlled systems.

**Figure 22:**
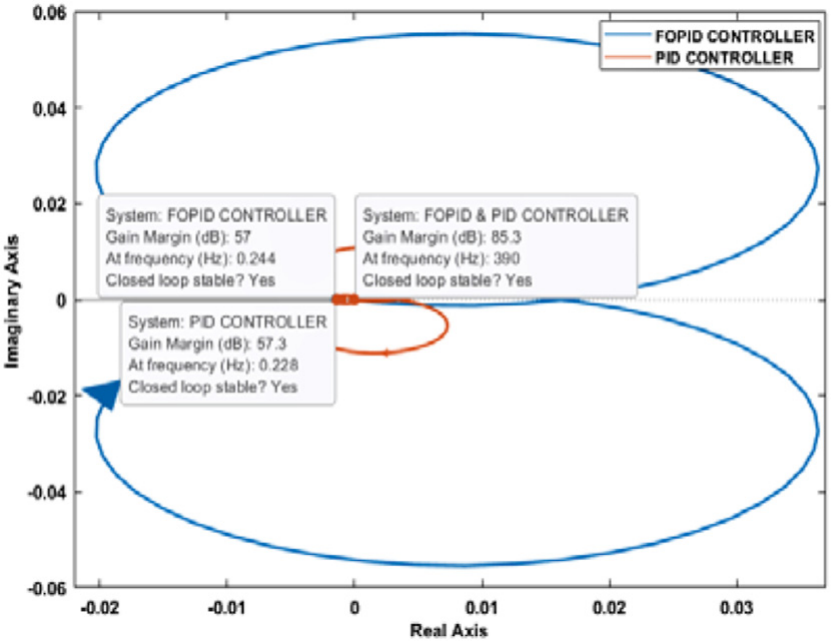
Nyquist plot of PID and FOPID-controlled Systems with Data Tip.

Figures 21 and 22 depict the Nyquist plot for the PID and FOPID-controlled systems. For both cases, the critical point lies outside of the Nyquist contours. Moreover, the gain margins in all cases are greater than one, and the phase margins are positive. Therefore, it can be determined that the PID and FOPID-controlled systems are stable as per Nyquist stability criteria.

## 5 Conclusion and future work

Since nuclear-renewable integration is a new era toward decarbonized energy infrastructure and renewables are intermittent, assuring the reliability and resiliency of the integrated system would be one of the most prevalent challenges in the upcoming days. This paper designs and demonstrates PID and FOPID controllers that have been tuned using TLBO and ABC algorithms for minimizing frequency variation in a microgrid consisting of renewables, energy storage systems, and nuclear reactors. The sole purpose of the research is to reduce the influence of frequency and power variations on microgrid operation when renewable energy sources, nuclear, and storage systems are present. The research objective is successfully attained through software simulations and comprehensive analyses. The time domain and frequency domain analysis indicate that both metaheuristic algorithms successfully tuned the PID and FOPID controllers for the N-R system. The TLBO optimization technique indeed validates the research findings obtained in the ABC optimization algorithm. Both the PID and FOPID controllers are compatible with N-R integrated systems. However, FOPID is a more responsive controller compared to PID.

Despite showing significant promise to handle a variety of optimization issues, ABC and TLBO have issues with premature convergence, memory capacity, and enormous iteration load. Although it is possible to achieve better results using other optimization techniques, those algorithms may have problems with local minimum stagnation and trouble choosing the suitable parameters.

The research considers baseload nuclear reactors. Therefore, frequency regulation in a load-following nuclear reactor in an N-R system can be a future research scope. Moreover, a Probabilistic Risk Assessment (PRA) is required for this kind of study since the system is entirely based on theoretical aspects and simulations. A PRA assessment can check the stability and impeccability of a system. A licensing document is also required for N-R integrated systems to bring the design into reality since no prior rules and regulations documentation is currently available for N-R systems. Moreover, robustness evaluation/sensitivity analysis should be conducted in the future study to assess the system performance’s uncertainty.

## Declarations

### Author contribution statement

Riyad Hasan; Shafakat Masud; Nawar Haque; Muhammad R. Abdussami: Conceived and designed the experiments; Performed the experiments; Analyzed and interpreted the data; Contributed reagents, materials, analysis tools or data; Wrote the paper.

### Funding statement

This research did not receive any specific grant from funding agencies in the public, commercial, or not-for-profit sectors.

### Data availability statement

Data included in article/supp. material/referenced in article.

### Declaration of interest’s statement

The authors declare no conflict of interest.

### Additional information

No additional information is available for this paper.

## Figures and Tables

**Table:** 

BESS	Battery Energy Storage System	N-R	Nuclear- Renewable
FESS	Flywheel Energy Storage System	N-R HES	Nuclear-Renewable Hybrid Energy System
PID	Proportional Integral Derivative	N-R MHES	Nuclear-Renewable Micro Hybrid Energy System
FOPID	Fractional Order Proportional Integral Derivative	PV	Photovoltaic
ABC	Artificial Bee Colony	RES	Renewable Energy Source
TLBO	Teaching Learning Based Optimization	WT	Wind Turbine
NPP	Nuclear Power Plant		

**Table:** 

Tin	0.04	T_LP2_	5
Tin/C	0.004	T_LP3_	10
T_WTG_	1.5	T_RH2_	9
K_HP_	2	T_BESS_	0.1
T_HP_	0.5	T_FESS_	0.01
T_GN_	0.08	K_MIC_	120
T_LP_	0.05	T_MIC_	20
T_RH1_	7	T_FC_	0.26

**Figure:**